# Supplementary material for: Plant nutation relies on steady propagation of spatially asymmetric growth pattern
Source: Quant Plant Biol. 2025 Jul 1;6:e30. doi: 10.1017/qpb.2025.10013 (PMC12451244; doi:10.1017/qpb.2025.10013)
Supplement: Rivière et al. supplementary material [file S2632882825100131sup001.zip › SM_resubmission250506_compressed.pdf]

Supplementary Information for
Plant nutation relies on steady propagation of spatially asymmetric growth pattern

Mathieu Rivière, Alexis Peaucelle, Julien Derr and Stéphane Douady
correspondence: julien.derr@ens-lyon.fr

This PDF file includes:

- Supplementary methods
- Supplementary text
- Figures S1 to S7
- Tables S1 to S2
- Legend for Movie S1
- References

Other supplementary materials for this manuscript include the following:

- Movie S1

Supplementary methods

Atomic force microscopy assays

Studied leaves were filmed overnight before the AFM experiment in the same conditions than for our kinematics experiments. Leaves were cut when $\phi \simeq 0^o$, when the difference in the elongation rates of the lateral faces is expected to reach a maxima. Several samples were taken free-handedly from the nutation zone with a razor blade. We kept track of the orientation of samples by resorting to several anatomical clues. The distal/apical axis was checked thanks to fact that trichomes consistently point toward the apex. The adaxial/abaxial axis was checked thanks to the bilateral symmetry of the rachis (see Fig. S6). Samples were then placed vertically on a microscopy slide and partially embedded in agarose, following a previously published protocol[3]. Samples were then kept immersed for the whole experiment in a solution of mannitol for plasmolysis. Measurements began after 20–30 min so that plasmolysis was reached (see Fig. S7). Identation was achieved with cantilevers with a spherical bead of diameter $25\ \mu m$. The indentation depth is not directly controllable but was generally comprised between $1\mu m$ and $5\mu m$. Force-distance curves were analyzed with the proprietary JPK software to extract Young moduli with Hertz contact model. We chose to work with relative Young moduli (normalized by the average Young moduli of the sample). The AFM mapping of the the sample was achieved by repeatedly indentating over a $100\ \mu m \times 100\ \mu m$ probing zone, which was manually moved to cover the regions of interest. Some aberrant points (impurities, trichomes) were withdrawn thanks to simple filters on relative height and stiffness values. Measurements outside falling outside the surface of the rachis surface were manually masked.

Immunolabelling experiments

Samples were taken as described in the previous section. Then, we performed multitarget immunohistochemistry following the recently published protocol [1]. The following antibodies were used: 2F4, JIM7, LM20, CBM3, CBM4, LM24 and SK1000. These bind respectively: pectins with low degree of methylesterification (DM), high DM pectins, high DM pectins, crystalline cellulose, amorphous cellulose, xyloglucans and mannans. The additional CBM4* treatment uses CBM4 antibodies after treating the samples with pectolyases to free the cellulose epitopes. Observations were made under a confocal microscope and we used the natural autofluoresnce signal of *Averrhoa carambola*'s cell walls observed at $\lambda = 405\ nm$ as a reference signal. Raw confocal images were processed with the ImageJ software. Each z-stack was reduced to a simple multichannel image with a maximum intensity projection. The transmission channel was used to discriminate the tissues of interest. For each sample, the peripheral tissues (excluding the central pith, vessels and outer epidermis) were divided in two arc-shaped regions according to the bilateral symmetry of the rachis (see Fig. S6). The intensity of the multiple fluorescence signals were averaged over these regions of interest. Finally, a contrast score was computed for each signal and for each sample: $(\langle I_{out}\rangle - \langle I_{in}\rangle) / (\langle I_{out}\rangle + \langle I_{in}\rangle)$, where I_X stands for the fluorescence intensity of either side.

Supplementary text

Estimating the envelope of a real-valued signal

A classical method to retrieve the instantaneous envelope of a real signal $s(t)$ is to compute the magnitude of its analytic representation $s_a(t) = s(t) + i \cdot \hat{s}(t)$, where $\hat{s}(t)$ is the Hilbert transform of s. The Hilbert transform $\hat{s}(t)$ is defined as the convolution product between $s(t)$ and the Cauchy kernel $\frac{1}{\pi t}$. For more details on this method, please refer to [2].

Details on the kinematic model of nutation

Projected elongation

Nutation causes the local orientation $\phi(t)$ of the rachis to vary over time. As a consequence, the elongation measured by a camera placed parallel to the immobile part is affected by geometric artifacts. Instead of measuring the actual total elongation rate of a face $\dot{\varepsilon}$, we get an apparent, projected elongation rate $\dot{\varepsilon}_\perp$ (see Fig. S2A). The projected elongation rate is defined as:

$$\dot{\varepsilon}_\perp = \frac{1}{l_\perp}\frac{\delta l_\perp}{\delta t} \tag{1}$$

where $l_\perp$ is the projected length of the considered element. A Taylor expansion at order 1 in δt gives the following approximation:

$$\dot{\varepsilon}_\perp = \dot{\varepsilon} - \dot{\phi}\tan\phi \tag{2}$$

Consider a non-elongating rod ($\dot{\varepsilon} = 0$) oscillating with angular frequency ω: $\phi(t) = \Delta\phi\cos\omega t$ (see Fig. S2B). Then, $\dot{\varepsilon}_\perp = \dot{\phi}\tan\phi$ oscillates with angular frequency 2ω (see Fig. S2C). Non-zero elongation rates simply modulate the average value of $\dot{\varepsilon}_\perp$.

Amplitude and localization of second first and second harmonic terms

In the non-trivial case where $\dot{\varepsilon} \neq 0$, $\dot{\delta} \neq 0$, the total projected elongation rate for a given face of the rachis is written as $\dot{\varepsilon}_\perp^{tot} = (\dot{\varepsilon} \pm \dot{\delta}) - \dot{\phi}\tan\phi$. In the limit of small angles:

$$\dot{\varepsilon}_\perp^{tot} \approx (\dot{\varepsilon} \pm \dot{\delta}) - \dot{\phi}\phi \tag{3}$$

Equation (3) immediately shows that there is an additional oscillating term at frequency ω since $\dot{\delta} \propto \sin\omega t$. It can further be shown that the $\dot{\phi}\phi$ term is responsible for the second harmonic contribution. Indeed, in the limit of $R\kappa \ll 1$, $\dot{\kappa} \approx \dot{\delta}/R$, thus:

$$\kappa(s_a, t) \approx -\frac{\dot{\delta}_0}{R\omega}\left[1 - \tanh^2\left(\frac{s_a - L_{gz}}{\Delta L}\right)\right]\cos\omega t \tag{4}$$

Then, $\phi = \int \kappa \mathrm{ds}$, thus:

$$\phi(s_a, t) \approx -\frac{\dot{\delta}_0}{R\omega}\int_{L_{tot}}^{s_a}\left[1 - \tanh^2\left(\frac{s'_a - L_{gz}}{\Delta L}\right)\right]\mathrm{ds'_a} \cdot \cos\omega \mathrm{t} \tag{5}$$

$$\approx \frac{\dot{\delta}_0 \Delta L}{R\omega}\left[\tanh\left(\frac{L_{tot} - L_{gz}}{\Delta L}\right) - \tanh\left(\frac{s'_a - L_{gz}}{\Delta L}\right)\right]\cos\omega t \tag{6}$$

Leading to the second harmonic of the nutation frequency, in the limit of $L_{tot} \gg L_{gz}$:

$$\dot{\phi}\phi \approx -\frac{1}{2}\left(\frac{\dot{\delta}_0 \Delta L}{R\omega}\right)^2 \omega\left[1 - \tanh\left(\frac{s'_a - L_{gz}}{\Delta L}\right)\right]^2 \sin 2\omega t \tag{7}$$

Finally, given the expressions of $\dot{\delta}$ and $\dot{\phi}\phi$, the second mode 2ω is expected to the strongest at the apical end of the growing plant, while the fundamental mode is expected to be strongest around $s_a = L_{gz}$.

Details on the fitting procedure for $\dot{E}$ and $\dot{D}$

The coarse elongation rate $\dot{E}$ and the coarse differential elongation rate $\dot{D}$ discussed in the Results section, and shown in Fig. 3 have been fitted to the following functions:

$$\dot{E}(s) = \frac{\dot{E}_0}{2}\left(1 - \tanh\left(\frac{s_a - L_{gz}}{\Delta L}\right)\right) \tag{8}$$

$$\dot{D}(s) = \dot{D}_0\left(1 - \tanh^2\left(\frac{s_a - L_{gz}}{\Delta L}\right)\right) \tag{9}$$

where s_a is the arc length defined from the apex, L_{gz} is the length of the growth zone and ΔL is the typical length scale of variation of the elongation rate. The $\dot{D}$ function here is proportional to $\partial_s \dot{E}$. The two functions were fitted together to the experimental data, in a single process with shared parameters L_{gz} and ΔL. The amplitudes of the functions were left independent of each other.

Probing cell wall elasticity during growth

We first measured the relative stiffness of the cell-wall in rachis section. The global mapping of a transverse cut reveals the extent of the rigidity variability across the inner tissues (see Fig. **S4**A). The relative stiffness (normalized by the global average) is widely distributed with a standard deviation of 52%. To further check if the lateral faces of the rachis had different mechanical properties, we focused our mapping effort on the peripheral tissues—with thicker walls—where we would expect maximum differences. We quantified the distributions of relative stiffness of 3 different growing leaves in the nutation region (see Fig. **S4**B). Our results show that the tissues on the inner face of the rachis are on average 17% softer (standard deviation 6%) than the tissues on the outer face. Among the three first moments of the relative rigidity distributions, only the mean gave a consistent trend across biological repetitions (see Fig. **S4** and Table **S1** for repetitions and complete distribution characterizations)

Probing cell wall composition changes during growth

We checked if the measured mechanical asymmetry correlates with biochemical asymmetry within the cell walls. We used multitarget immunolabelling to probe epitope asymmetry across the tissue (see Fig. **S5**). We targeted several important families of components of the cell wall: cellulose, hemicelluloses and homogalacturonan domains of pectins. Additionally, the auto-fluorescence signal of *A. carambola* cell walls was used as a reference as it allows to check for any underlying anatomical asymmetry such as cell wall thickness for example. Results in Fig. **S5**B show the distributions of signal contrast for the different tested antibodies. Student's t-test revealed no statistically significant bias in the auto-fluorescence signal ($p = 0.13 > 5 \times 10^{-2}$, see Fig. **S5**D). We then conducted a systematical comparison of antibody fluorescence and auto-fluorescence contrasts via Welch's unequal variances t-test. Our results show no statistically significant shift from the reference signal for crystalline cellulose (CBM3), amorphous cellulose on pectolyase-treated samples (CBM4*) and mannans (SK1000). Xyloglucans (LM24) and regular amorphous cellulose treatments (CBM4) returned p-values of 1.3×10^{-2} and 2.8×10^{-2}, advocating for a slight but unconclusive signal asymmetry. In contrast, both 2F4 and LM20 signals, marking HG with low and high degrees of methylesterification respectively, did significantly differ from the auto-fluorescence signal (both $p < 5 \times 10^{-5}$ – for a complete description of the statistical tests results, please refer to Table **S2**). From the fluorescence contrasts, we see that the 2F4 and LM20 signals are on average 5% and 13% stronger on the outer half of the rachis than on its inner half.

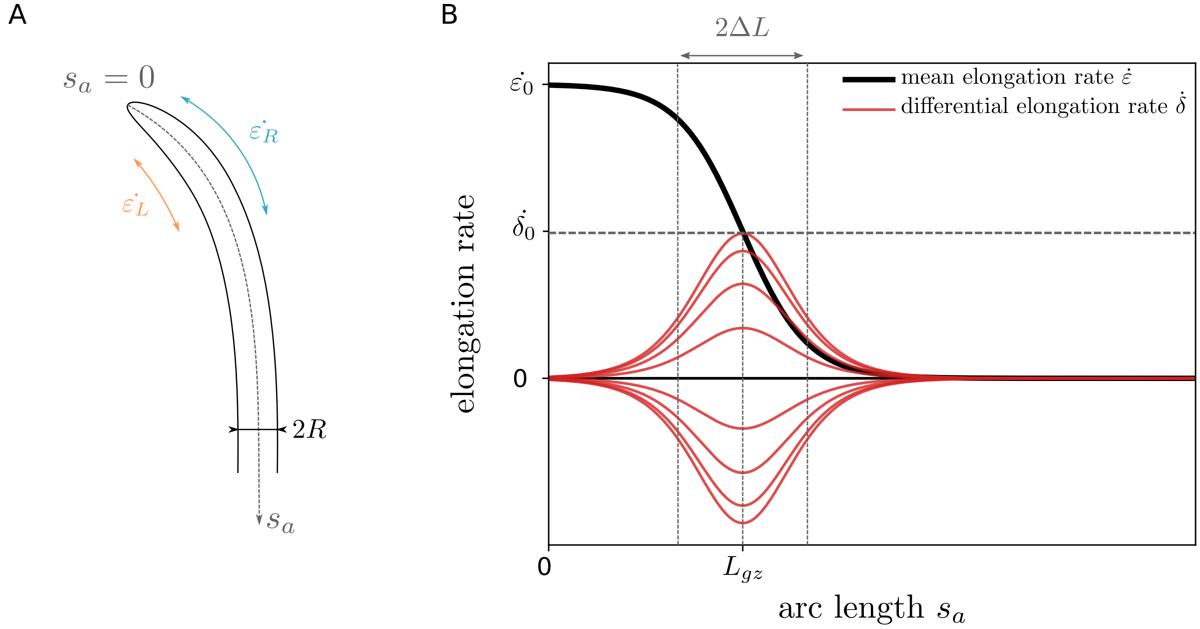

Figure S1: **Details on the kinematic model for nutation.** (A) Geometrical parametrization of the model. (B) Elongation and differential elongation laws. The elongation rate $\dot{\varepsilon}$ shows a growth zone of length L_{gz} defined from the apex. The differential elongation rate $\dot{\delta}$ takes place where elongation is dropping. It is proportional to the spatial derivative of $\dot{\varepsilon}$. The differential elongation is furthermore modulated in time by a sine function of angular frequency ω. Both functions are defined with hyperbolic functions, as discussed in the Methods section. Both quantities have a characteristic length of variation ΔL.

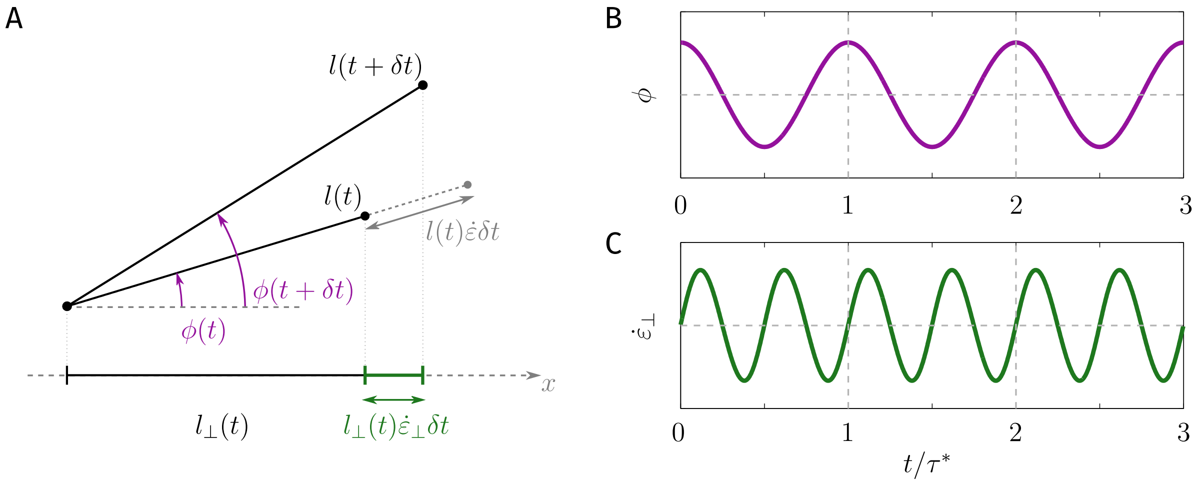

Figure S2: **Apparent elongation of a rod undergoing nutation in the simple no-curvature case.** (A) A rod of length $l(t)$ elongates at a rate $\dot{\varepsilon}$ while its angle ϕ with the (Ox) axis varies. The apparent elongation $\dot{\varepsilon}_\perp$ measured along (Ox) is the elongation of the orthogonal projection of the rod $l_\perp(t)$. (B) Periodic variations of ϕ in time due to nutation of period τ^*. (C) Apparent elongation $\dot{\varepsilon}_\perp$ in time. The frequency of $\dot{\varepsilon}_\perp$ is double the nutation frequency.

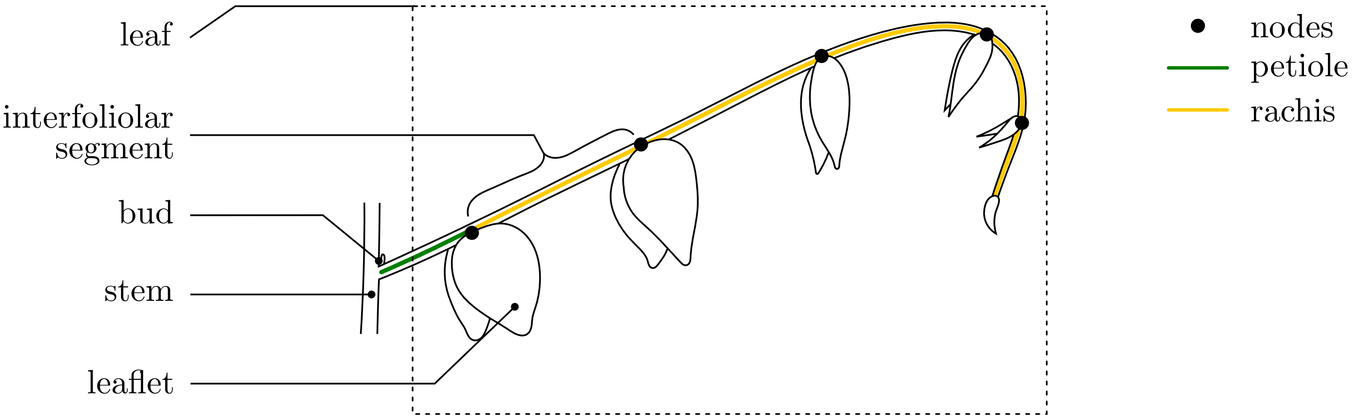

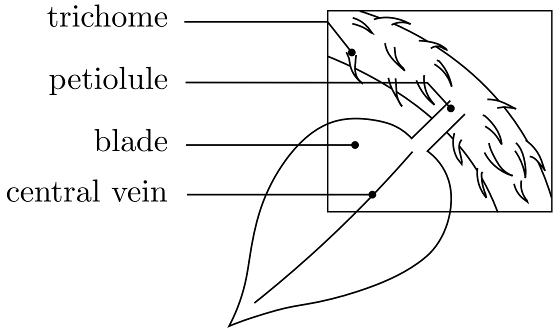

Figure S3: **Drawing of a growing Averrhoa carambola compound leaf.** The top panel represents the entirety of a compound leaf and details the associated vocabulary. The bottom panel is a close-up around a leaflet, with additional details and anatomical vocabulary. The unit of interest for this study is the rachis, which can be seen as the "center vein" of the compound leaf ; and its sub-units: the interfoliolar segments, separated by nodes where leaflets are connected to the rachis.

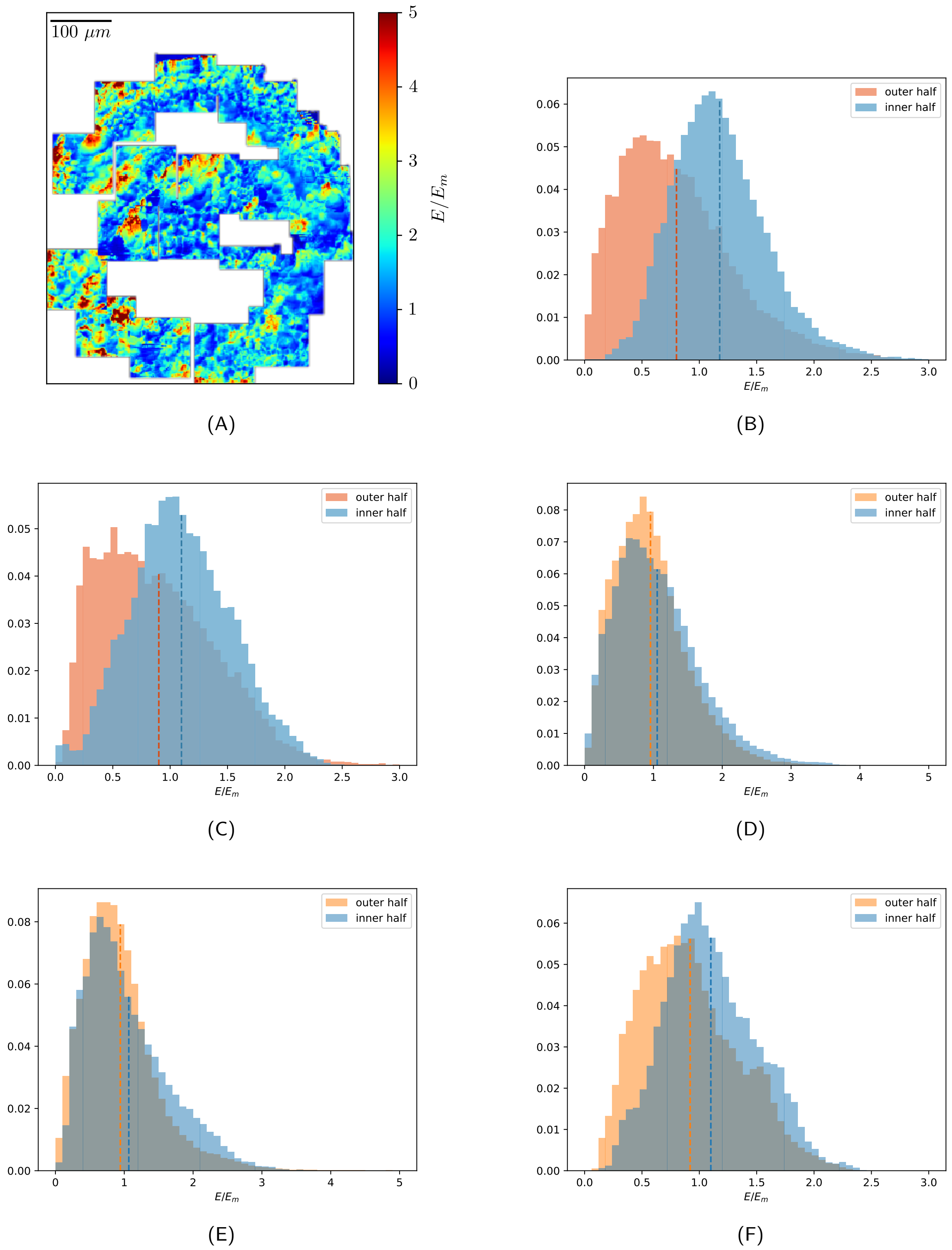

Figure S4: **Distributions of relative stiffnesses in a cross-section of the rachis measured by AFM.** (A) Example of a stiffness map of the rachis with partial mapping of the central pith. (B-F) Histograms of the convex/outer (orange) and concave/inner (blue) faces of the rachis are drawn separately for comparison. The dotted lines correspond to the means. (B-C) Two samples from rachis n0. (D-E) Two samples from rachis n1. (F) A single sample from rachis n2.

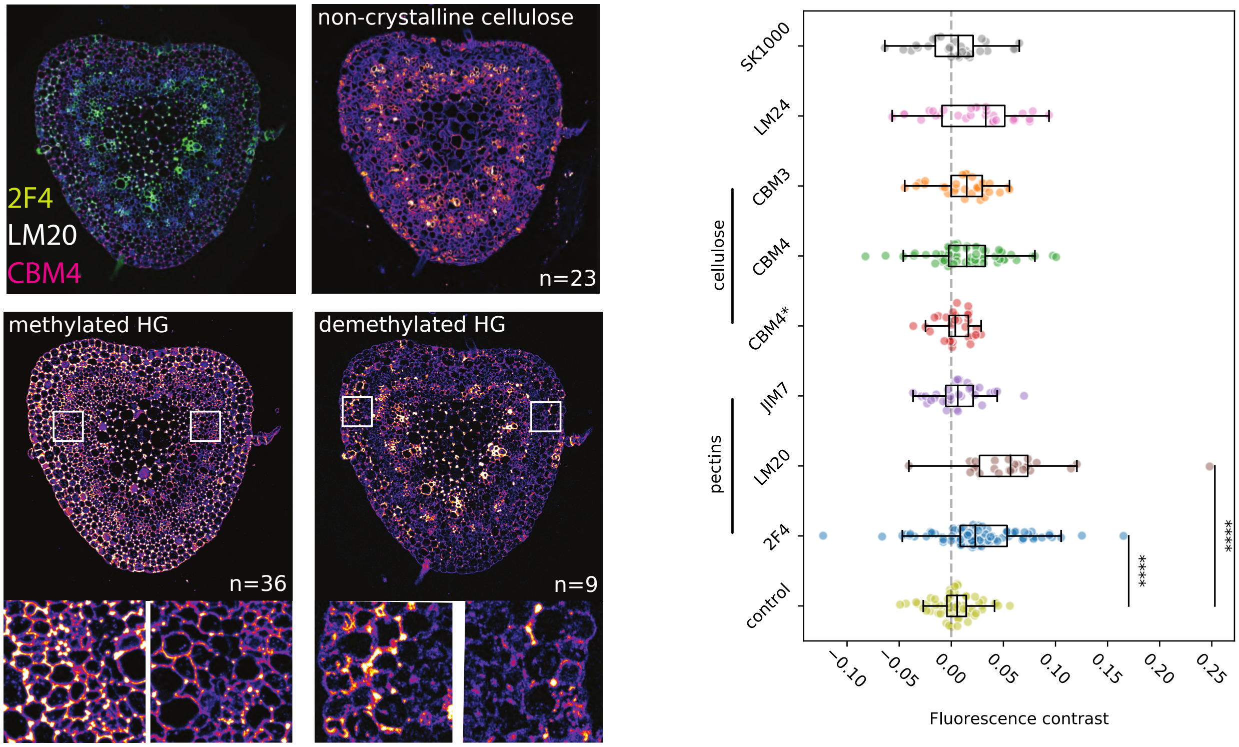

(A) (B)

Figure S5: **Quantification of the biochemical asymmetry of cell-walls across the rachis within the nutation zone.** **(A)** Fluorescence images of cross sections from a single rachis in the nutation zone. Three histochemical treatments are presented together (composite image) and individually. The used antibodies target non-crystalline cellulose (CBM4) and homogalacturonans with a low/high methyl-esterification degree (2F4/LM20 respectively). For the HG images, two zoomed windows are presented for a detailed comparison of the lateral faces of the rachis. **(B)** Distributions of fluorescence contrast for several immunolabelling treatments. The contrast is calculated as $(< I_{out} > - < I_{in} >)/(< I_{out} > + < I_{in} >)$, where $< I_X >$ corresponds to the fluorescence intensity averaged over regions of interest located either on the inner or outer half of the rachis. Each dot represents a single measurement. The distributions are overlayed with their respective boxplot. Significant Student's t-test have been indicated. The control measurement corresponds to the natural autofluorescence signal of *Averrhoa carambola*'s cell walls observed at $\lambda = 405\ nm$.

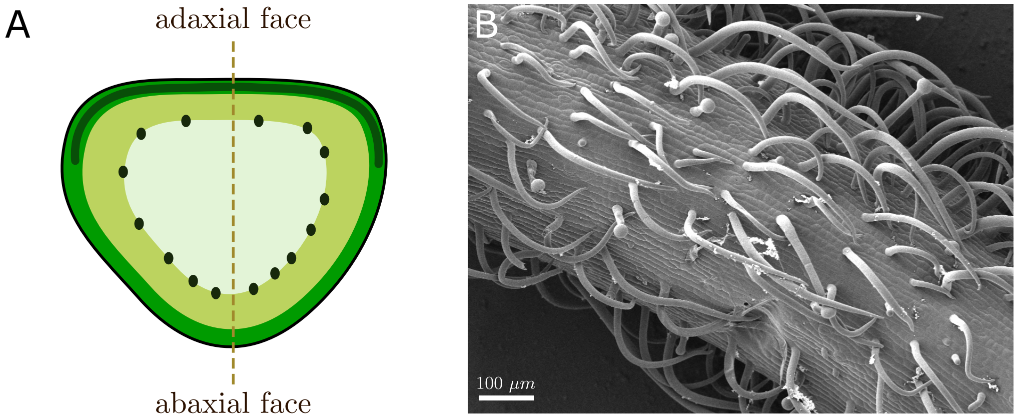

Figure S6: **Useful clues to determine the orientation of a sample**. (A) Simplified picture of a cross-section of *Averrhoa carambola* rachis. The bilateral symmetry of the rachis allows a clear distinction between the lateral faces and the abaxial/adaxial faces. Flatness, chloroplast and vessel densities allow to distinguish the adaxial and abaxial faces from one another. (B) Cryo-SEM image of a carambola rachis. The rachis is covered with trichomes that all point toward the apex

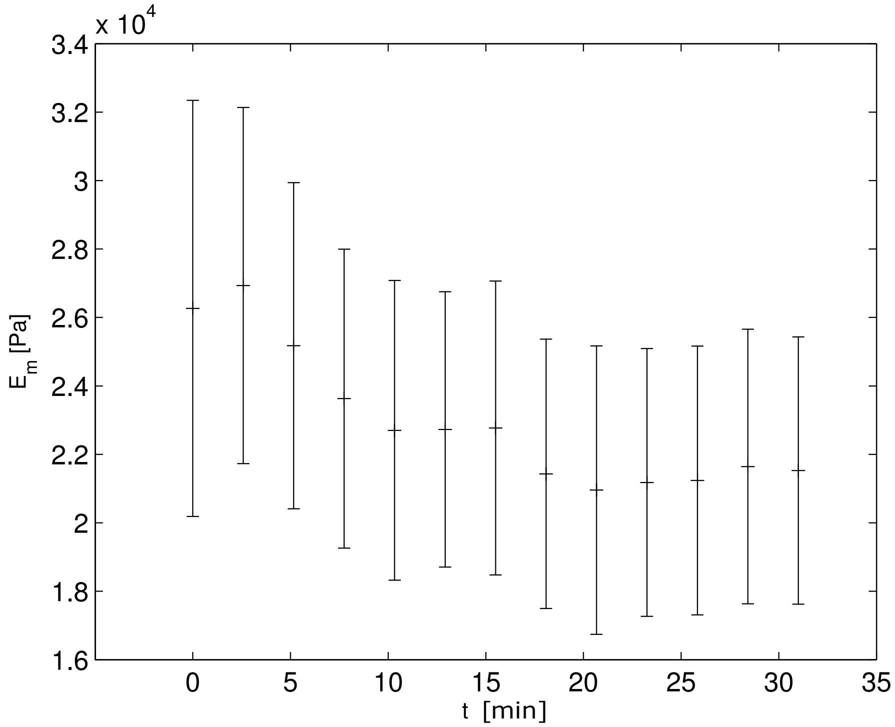

Figure S7: **Plasmolysis dynamics and plasmolysis effects on Atomic Force Microscopy measurements.** A sample is immersed in a mannitol solution and a subregion of this sample is mapped by AFM every 2.5 min. The evolution of the Young modulus averaged over the entire subregion E_m is shown here. We see that after 20 min, the value of E_m stabilizes. Error bars represent the standard deviation over the AFM map. This experiment was conducted for a single sample.

Figure	Plant	Sample	μ_{in}	μ_{out}	$1-\mu_{\mathrm{out}}/\mu_{\mathrm{in}}$	σ_{in}	σ_{ost}	$1-\sigma_{\mathrm{out}}/\sigma_{\mathrm{in}}$	γ_{in}	γ_{out}	$1-\gamma_{\mathrm{out}}/\gamma_{\mathrm{in}}$
Fig. **S4**B	n0	1	1.10	0.90	$1.80-10^{-1}$	0.45	0.56	$-2.87\cdot10^{-1}$	0.16	1.77	-9.99
Fig. **S4**C		2	1.18	0.80	$5.20-10^{-1}$	0.42	0.49	$-1.75\cdot10^{-1}$	0.75	0.91	$-2.01\cdot10^{-1}$
Fig. **S4**D	n1	1	1.05	0.96	$9.26-10^{-2}$	0.61	0.54	$1.13\cdot10^{-1}$	0.87	1.95	-1.21
Fig. **S4**E		2	1.07	0.94	$1.16-10^{-1}$	0.87	0.71	$1.85\cdot10^{-1}$	12.95	10.28	$2.06-10^{-1}$
Fig. **S4**F	n2	1	1.10	0.92	$1.84-10^{-1}$	0.40	0.42	$-5.72\cdot10^{-2}$	0.32	0.50	$-5.46-10^{-1}$

Table S1: Summary of the quantification of the three first moments of the relative stiffness distributions.

The experiment was replicated on three single rachis of three different plants (n0, n1 and n2). For each plant/rachis, the different samples correspond to different transverse sections. Subscripts *in* and *out* correspond to the inner (or concave) and outer (or convex) faces of the rachis, respectively. Remember that our working hypothesis is that, as the rachis bends, its outer face grows faster than the outer one.

	avg contrast	std contrast	Student's t-test t-value	p-value	Welch's t-test t-value	p-value	N
2F4	2.78×10^{-2}	4.22×10^{-2}	6.17	2×10^{-8}	-4.55	1.18×10^{-5}	89
CBM3	1.29×10^{-2}	2.51×10^{-2}	2.8	8.75×10^{-3}	-1.67	0.1	31
CBM4	1.58×10^{-2}	3.46×10^{-2}	3.46	1.04×10^{-3}	-2.23	2.79×10^{-2}	58
CBM4*	4.04×10^{-3}	1.56×10^{-2}	1.35	0.19	-3.33×10^{-3}	1	28
JIM7	7.18×10^{-3}	2.30×10^{-2}	1.74	9.18×10^{-2}	-0.64	0.52	32
LM20	6.00×10^{-2}	5.03×10^{-2}	5.84	5.04×10^{-6}	-5.28	1.41×10^{-5}	25
LM24	2.37×10^{-2}	3.91×10^{-2}	3.32	2.35×10^{-3}	-2.59	1.35×10^{-2}	31
SK1000	4.73×10^{-3}	3.12×10^{-2}	0.79	0.44	-0.11	0.91	28
control	4.02×10^{-3}	2.05×10^{-2}	1.52	0.13			61

Table S2: Summary of the statistical tests applied to immunolabelling experiments.

Here, N corresponds to the total number of different samples tested. These samples are taken from 2 independant biological repetitions, on the interfoliolar segment where nutation occurs and the the two adjacent ones.

Legends for other supplementary material not included in this file:

Movie S1: Fourty-eight hours of development of an *Averrhoa carambola* compound leaf shown through synchronized top and side views. Nutation, the swinging motion of the rachis, is most easily observed in the top-view. The side-view offers a complete vision of growth motions of a compound leaf, with the typical hook shape and unfurling motion. Scale bars = 1 cm.

References

[1] Kalina T Haas et al. "Multitarget immunohistochemistry for confocal and super-resolution imaging of plant cell wall polysaccharides". In: *Bio-protocol* 10.19 (2020), e3783–e3783.

[2] TG Kincaid. "The complex representation of signals". In: *TIS R67# MH5, General Electric Co* (1966).

[3] Alexis Peaucelle. "AFM-based mapping of the elastic properties of cell walls: at tissue, cellular, and subcellular resolutions". In: *Journal of visualized experiments: JoVE* 89 (2014).
